# Disulfide high mobility group box-1 causes bladder pain through bladder Toll-like receptor 4

**DOI:** 10.1186/s12899-017-0032-9

**Published:** 2017-05-25

**Authors:** Fei Ma, Dimitrios E. Kouzoukas, Katherine L. Meyer-Siegler, Karin N. Westlund, David E. Hunt, Pedro L. Vera

**Affiliations:** 1 Research and Development, Lexington Veterans Affairs Medical Center, 1101 Veterans Drive, Room C-327, Lexington, Kentucky 40502 USA; 2 Department of Physiology, University of Kentucky, Lexington, Kentucky USA; 3 Saha Cardiovascular Research Center, University of Kentucky, Lexington, Kentucky USA; 4 Department of Natural Sciences, St. Petersburg College, St. Petersburg, Florida USA; 5 Department of Surgery, University of Kentucky, Lexington, Kentucky USA; 6 Present Address: Department of Molecular Pharmacology and Therapeutics, Loyola University Chicago, Maywood, Illinois USA

**Keywords:** HMGB1, TLR4, RAGE, bladder pain, abdominal mechanical hypersensitivity, urothelium

## Abstract

**Background:**

Bladder pain is a prominent symptom in several urological conditions (e.g. infection, painful bladder syndrome/interstitial cystitis, cancer). Understanding the mechanism of bladder pain is important, particularly when the pain is not accompanied by bladder pathology. Stimulation of protease activated receptor 4 (PAR4) in the urothelium results in bladder pain through release of urothelial high mobility group box-1 (HMGB1). HGMB1 has two functionally active redox states (disulfide and all-thiol) and it is not known which form elicits bladder pain. Therefore, we investigated whether intravesical administration of specific HMGB1 redox forms caused abdominal mechanical hypersensitivity, micturition changes, and bladder inflammation in female C57BL/6 mice 24 hours post-administration. Moreover, we determined which of the specific HMGB1 receptors, Toll-like receptor 4 (TLR4) or receptor for advanced glycation end products (RAGE), mediate HMGB1-induced changes.

**Results:**

Disulfide HMGB1 elicited abdominal mechanical hypersensitivity 24 hours after intravesical (5, 10, 20 μg/150 μl) instillation. In contrast, all-thiol HMGB1 did not produce abdominal mechanical hypersensitivity in any of the doses tested (1, 2, 5, 10, 20 μg/150 μl). Both HMGB1 redox forms caused micturition changes only at the highest dose tested (20 μg/150 μl) while eliciting mild bladder edema and reactive changes at all doses. We subsequently tested whether the effects of intravesical disulfide HMGB1 (10 μg/150 μl; a dose that did not produce inflammation) were prevented by systemic (i.p.) or local (intravesical) administration of either a TLR4 antagonist (TAK-242) or a RAGE antagonist (FPS-ZM1). Systemic administration of either TAK-242 (3 mg/kg) or FPS-ZM1 (10 mg/kg) prevented HMGB1 induced abdominal mechanical hypersensitivity while only intravesical TLR4 antagonist pretreatment (1.5 mg/ml; not RAGE) had this effect.

**Conclusions:**

The disulfide form of HMGB1 mediates bladder pain directly (not secondary to inflammation or injury) through activation of TLR4 receptors in the bladder. Thus, TLR4 receptors are a specific local target for bladder pain.

## Background

Common causes of bladder pain are bacterial infection, painful bladder syndrome/interstitial cystitis (PBS/IC) and cancer. Bladder pain in the absence of infection or bladder pathology is a feature of PBS/IC patients, along with increased frequency and urgency [1]. However, common rodent models of bladder pain usually produce significant bladder injury and inflammation [2, 3].

Cyclophosphamide (CYP)-induced cystitis (a widely used chemical model) elicits severe bladder inflammation and urothelial damage along with significantly decreased abdominal mechanical threshold [4, 5]. Interestingly, CYP-induced bladder pain (abdominal mechanical hypersensitivity) was blocked by systemic administration of a high-mobility group box 1 protein (HMGB1) neutralizing antibody or a HMGB1 receptor antagonist without changing CYP-induced inflammation [5].

HMGB1 is a ubiquitous and abundant non-histone nuclear chromatin-binding protein and a damage-associated molecular pattern molecule. HMGB1 is actively secreted in response to inflammatory signals, acting as a pro-inflammatory molecule in addition to its passive release from necrotic cells in various organs [6]. The extracellular activities of HMGB1 depend on the redox state of HMGB1 resulting in activation of different HMGB1 receptors. Physical/chemical trauma to tissues or organs results in the release of all-thiol (all-reduced) HMGB1, which binds to receptor for advanced glycation end products (RAGE) and potentiates chemotaxis [7]. During inflammation, all-thiol HGMB1 may be oxidized to the disulfide form of HGMB1, which then binds to Toll-like receptor 4 (TLR4) to induce cytokine production [7]. It is likely that both redox forms contribute to inflammation resulting from tissue damage. HMGB1 is a key player in the extracellular environment as a pro-inflammatory molecule and is also gaining prominence as a mediator in pain processing [6, 8].

We recently reported that activation of urothelial protease activated receptor 4 (PAR4) elicits bladder pain in mice without causing overt bladder inflammation [9]. In this model, PAR4 activation results in release of urothelial macrophage migration inhibitory factor (MIF) [9] and HMGB1 [10] along with abdominal mechanical hypersensitivity, representative of bladder pain. Systemic pretreatment with MIF antagonist prevented urothelial HMGB1 release [9] and abdominal mechanical hypersensitivity caused by intravesical PAR4-activating peptide (PAR4-AP) [9]. Moreover, systemic administration of a HMGB1 inhibitor also blocked abdominal mechanical hypersensitivity caused by intravesical PAR4-AP [10]. This indicates that HMGB1 signaling is involved in PAR4-induced bladder pain. However, it is still not known which redox form of HMGB1 is responsible for bladder pain and the type or location of the HMGB1 receptor mediating the effect. The current study utilized two redox forms of HMGB1 and receptor-specific antagonists in a rodent model of bladder pain without inflammation to explore the etiology of bladder pain.

## Methods

### Animals

All animal experiments were approved by the Lexington Veterans Affairs Medical Center Institutional Animal Care and Use Committee (VER-11-016-HAF) and performed according to the guidelines of the National Institutes of Health.

### Disulfide or all-thiol HMGB1 treatment by intravesical instillation

13 – 17 week-old female C57BL/6 (SPF, 20-25 g, Jackson Laboratory, Bar Harbor, ME) were accommodated in ventilated animal housing with 14/10 light/dark cycle. Isoflurane-anesthetized mice were transurethrally catheterized (PE10, 11 mm length) and drained of urine. Disulfide HMGB1, all-thiol HMGB1 (1, 2, 5, 10 and 20 μg; 150 μl, HMGBiotech S.r.l., Milano, Italy) or vehicle control groups (PBS; 150 μl) (3-6/group) were randomly instilled into the bladder lumen and held for 1 hour [9, 10]. In other experiments mouse groups were pretreated with TLR4 antagonist TAK-242 (30 min prior) [11], or RAGE antagonist FPS-ZM1 (15 min prior) [12], either intraperitoneally (TAK-242, 3 mg/kg; FPS-ZM1, 10 mg/kg) or intravesically (TAK-242, 1.5 mg/ml; FPS-ZM1, 5 mg/ml). Then 10 μg disulfide HMGB1 was instilled and held for 1 hour as described.

### Abdominal mechanical hypersensitivity test

Abdominal mechanical hypersensitivity was tested in instilled mice as previously described [10]. Briefly, von Frey filaments of ascending bending force (0.008, 0.020 0.040, 0.070 g) were pressed to the lower abdominal region in trials of 10 before (baseline) and 24 hours after HMGB1 instillation. Positive response was defined as any one of three behaviors: 1) licking the abdomen, 2) flinching/jumping, or 3) abdomen withdrawal. Mice responding more than 30% to the weakest filament (0.008 g) during baseline testing were excluded from the study.

Awake mice were tested for abdominal mechanical hypersensitivity and micturition changes 24 hours after bladder instillation.

### Voided Stain on Paper (VSOP): micturition volume and frequency

Micturition volume and frequency were measured in mice using VSOP method [13]. Briefly, mice were gavaged with water (50 μl/g body weight) to induce diuresis, then placed in a plastic enclosure and allowed to move freely. Filter paper was placed under each mouse to collect urine during a 2-hour observation period. Micturition volumes were determined by linear regression using a set of known volumes. Micturition frequency was defined as the number of micturition within 2 hours.

### Histology

Bladders were removed under anesthesia, fixed in 10% formalin, embedded in paraffin for histology and mice were euthanized at the end of the experiment.

Paraffin sections (5 μm) were processed for routine hematoxylin and eosin (H&E) staining. H&E stained sections were evaluated by a pathologist blinded to the experimental treatment and scored separately for edema and inflammation according to the following scale: 0 = no edema; no infiltrating cells; 1 = mild submucosal edema; occasional inflammatory cells; 2 = moderate edema; several inflammatory cells; 3 = frank edema, vascular congestion; many inflammatory cells [10].

### Statistical analyses

Changes in positive response frequency (%) to von Frey stimulation at baseline and 24 hours after treatment were evaluated using a within subject 2-way (Time x Filament Strength) ANOVA. When the Time factor (pre *vs*. post) was significant, differences at each filament strength were compared (pre *vs.* post) using t-tests with a multiple comparison adjustment (Holm-Sidak) [14]. Single t-tests (mean = 0) were performed for the histological scores.

All data are presented as mean ± SE [14], with statistical differences of *p* ≤ 0.05 considered significant. All statistical analyses were performed using R [15].

## Results

### HMGB1 redox form elicits abdominal mechanical hypersensitivity

**Fig. 1:**
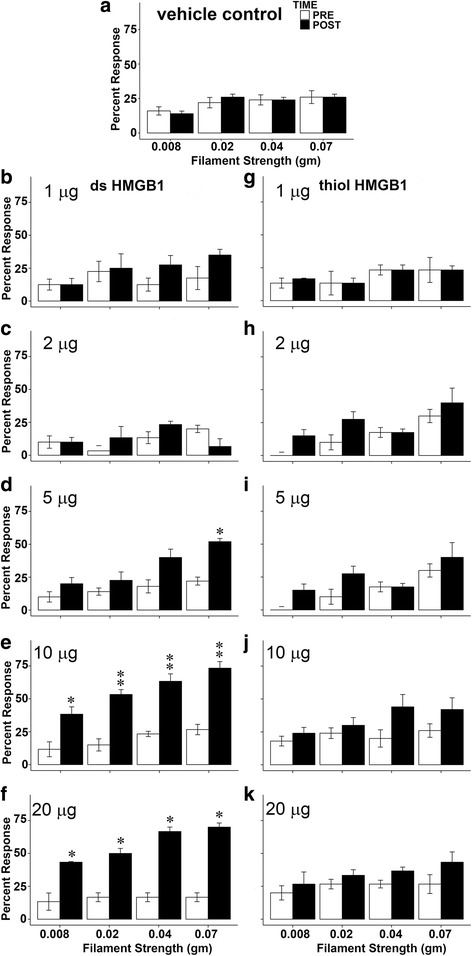
Disulfide and all-thiol HMGB1 dose response effects on abdominal mechanical thresholds. (**a**) vehicle control, (**b**) 1 μg (n = 4) and (**c**) 2 μg (n = 3) disulfide HMGB1 did not affect abdominal mechanical threshold. (**d**) 5 μg (n = 5) disulfide HMGB1 significantly increased abdominal sensitivity using a 0.07 g filament. (**e**) 10 μg (n = 6) and (**f**) 20 μg (n = 3) disulfide HMGB1 significantly induced abdominal hypersensitivity using all four von Frey filaments. None of the all-thiol HMGB1 doses (**g**) 1 μg (n = 3), (**h**) 2 μg (n = 3), (**i**) 5 μg (n = 4), (**j**) 10 μg (n = 5) and (**k**) 20 μg (n = 3) changed abdominal mechanical sensitivity. *p < 0.05, **p < 0.01 compared with pre-instillation

We measured responses to von Frey filaments applied to the abdominal/perineal area at baseline (before) and 24 hours after bladder HMGB1 instillation of either disulfide HMGB1 or all-thiol HMGB1 (1, 2, 5, 10 and 20 μg). Intravesical vehicle control, 1 or 2 μg of disulfide HMGB1 did not cause any abdominal mechanical hypersensitivity 24 hours after instillation (Fig. 1a-c). Figure 1d shows that 5 μg (n = 5) of disulfide HMGB1 resulted in significant mechanical hypersensitivity of abdominal/perineal area only with the highest filament tested (0.07 g; Fig. 1d). Higher doses of disulfide HMGB1 10 (n = 6) or 20 μg (n = 3) significantly increased von Frey responses compared to baseline for all filaments tested (Fig. 1e, f). In contrast, intravesical all-thiol HMGB1 did not cause any change in abdominal mechanical hypersensitivity for any of the doses tested (Fig. 1g-k).

### Micturition changes after HMGB1 bladder instillation

Table 1 shows micturition volume and frequency changes after different doses of either disulfide or all-thiol HMGB1. Only the highest dose of disulfide and all-thiol HMGB1 (20 μg, n = 3) resulted in a significant decrease in volume (141 ± 7 μl, 177 ± 24 μl) compared to vehicle control treated group (n = 5; 301 ± 39 μl). This dose of disulfide or all-thiol HMGB1 also increased micturition frequency (7.3 ± 0.7, 4.7 ± 0.3 *vs* 3.0 ± 0.4 of PBS). Lower doses of disulfide or all-thiol HMGB1 had no effect on these two micturition parameters (Table 1).

**Table 1:** Effects of intravesical disulfide and all-thiol HMGB1 on mouse micturition

	ds HMGB1		all-thiol HMGB1	
Dose (μg)	Volume (μl)	Freq	Volume (μl)	Freq
0	301 ± 39.3	3.0 ± 0.4	301 ± 39.3	3.0 ± 0.4
1	288 ± 20.1	3.5 ± 0.6	230 ± 9.2	4.3 ± 0.7
2	359 ± 13.9	2.0 ± 0.0	240 ± 79.8	5.3 ± 1.7
5	255 ± 36.8	3.2 ± 0.9	267 ± 35.0	4.3 ± 0.9
10	264 ± 25.5	4.1 ± 1.0	214 ± 15.8	4.0 ± 0.8
20	141 ± 7.3*	7.3 ± 0.7*	177 ± 23.7*	4.7 ± 0.3*

0 = vehicle control

*p < 0.05 compared with 0 μg disulfide HMGB1

### Histological changes

**Fig. 2:**
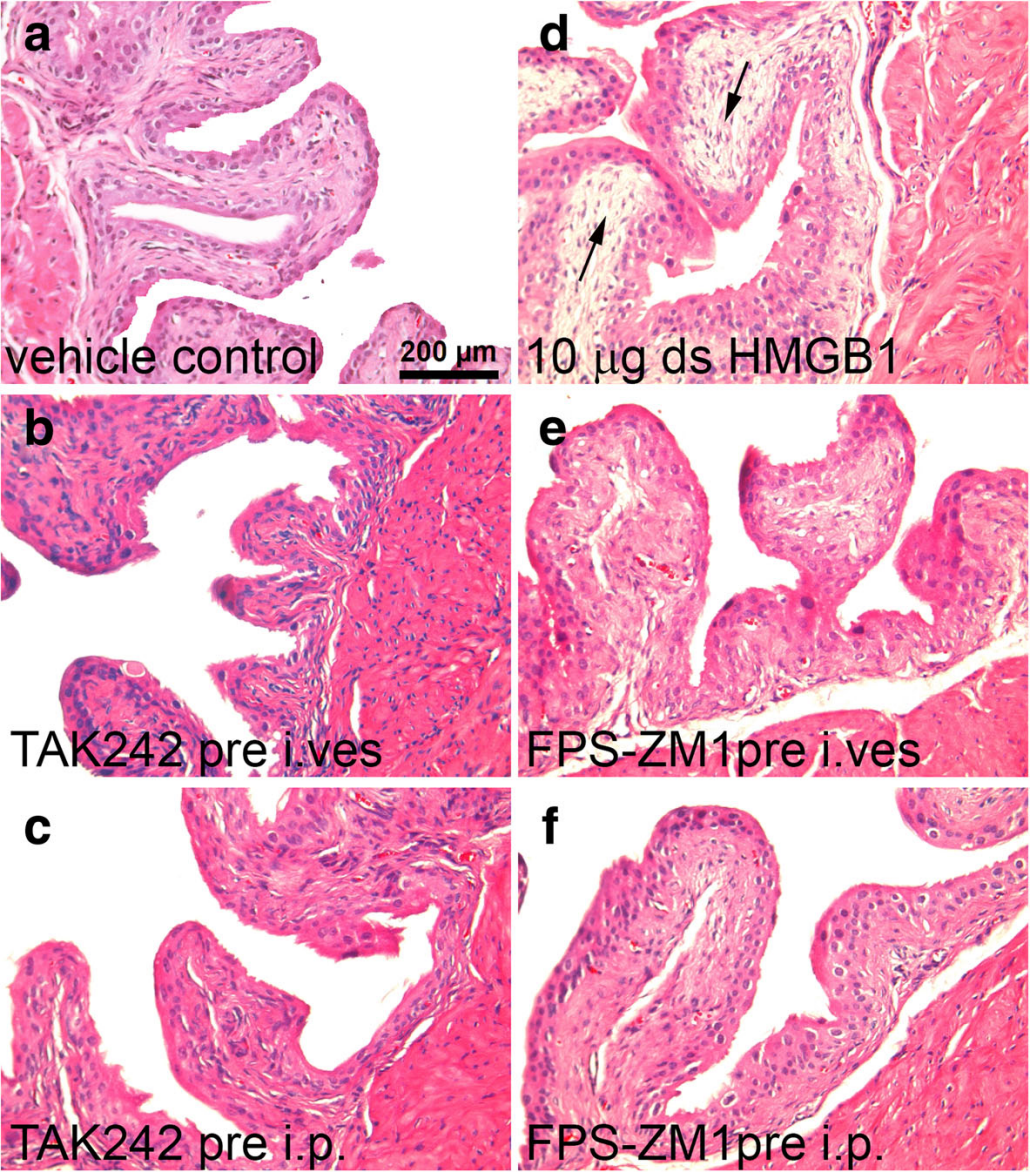
Bladder histology after disulfide HMGB1 and pretreatment with intravesical or intraperitoneal HMGB1 receptor antagonists. (**a**) Intravesical vehicle control instillation (n = 5), (**b**) intravesical TAK242 (n = 4), (**c**) intraperitoneal TLR4 antagonist TAK242 (n = 5), (**d**) 10 μg disulfide HMGB1 induced submucosal fibrosis with lamina propria expansion (black arrows) (n = 6), (**e**) intravesical FPS-ZM1 pretreatment (n = 6) or (**f**) intraperitoneal RAGE antagonist FPS-ZM1 (n = 3)

H&E stained bladder sections from mice that received different doses of disulfide HMGB1, all-thiol HMGB1 or vehicle control (PBS) were examined by a pathologist blinded to the treatment and scored for inflammation and edema changes (Table 2). Intravesical installation of vehicle control did not produce any inflammation or edema (Table 2). Disulfide or all-thiol HMGB1 at doses < 20 μg did not produce any inflammation while minimal inflammation (not statistically significant) was observed after 20 μg of either disulfide or all-thiol HMGB1. Either disulfide or all-thiol HMGB1 at all doses tested induced minimal to mild bladder edema and stromal reactive changes in some mice (reactive submucosal fibrosis with lamina propria expansion, Fig. 2d, Table 2), not statistically significant compared to vehicle control (Fig. 2a).

**Table 2:** Effects of intravesical disulfide and all-thiol HMGB1 on mouse bladder histology

	Inflammation		Edema	
Dose (μg)	ds HMGB1	all-thiol HMGB1	ds HMGB1	all-thiol HMGB1
0	0	0	0	0
1	0	0	0.4 ± 0.2	1.3 ± 0.7
2	0	0	0.7 ± 0.7	0.7 ± 0.3
5	0	0	0	1.0 ± 0.6
10	0	0	0.2 ± 0.2	0.9 ± 0.5
20	0.3 ± 0.3	0.3 ± 0.3	1.0 ± 1.0	0.7 ± 0.7

0 = vehicle control

### Effect of TLR4 and RAGE antagonism on disulfide HMGB1 induced hypersensitivity

**Fig. 3:**
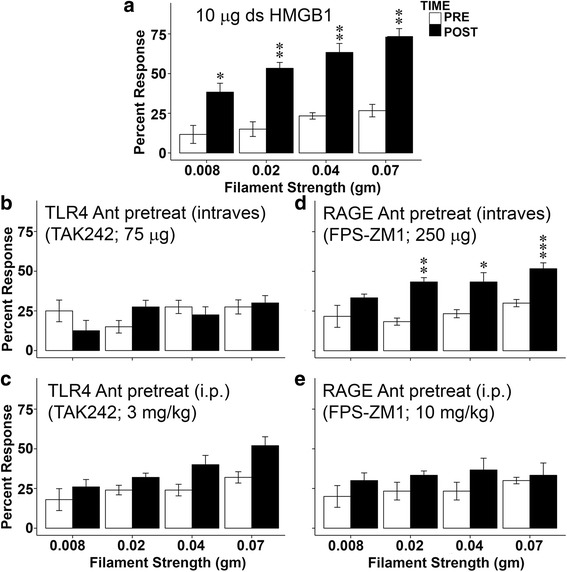
TLR4 or RAGE antagonist pretreatment prevented abdominal mechanical hypersensitivity induced disulfide HMGB1. (**a**) 10 μg disulfide HMGB1 significantly increased abdominal mechanical sensitivity (percent responses) using all four von Frey filaments (n = 6). (**b**) Intravesical TLR4 antagonist TAK242 blocked disulfide HMGB1 induced abdominal hypersensitivity (n = 4). (**c**) Intraperitoneal TAK242 reduced mechanical hypersensitivity induced by 10 μg disulfide HMGB1 (n = 5). (**d**) Intravesical infusion of RAGE antagonist PSF-ZM1, however, did not affect abdominal mechanical hypersensitivity induced by disulfide HMGB1 (n = 6). (**e**) Intraperitoneal injection of PSF-ZM1 prevented disulfide HMGB1 induced mechanical hypersensitivity (n = 3). *p < 0.05, **p < 0.01, ***p < 0.001 Twenty-four hours post-instillation compared with pre-instillation

We chose the first dose of disulfide HMGB1 that showed significantly increased abdominal mechanical sensitivity across all von Frey filaments (10 μg; Fig. 3a) without inflammation to test the effect of specific HMGB1 receptor antagonism. Pretreatment with specific TLR4 (TAK-242) or RAGE (FPS-ZM1) antagonists was used to investigate which receptor signaling mechanism was activated by intravesical disulfide HMGB1 resulting in increased abdominal mechanical hypersensitivity.

Intravesical pretreatment with TAK-242 (TLR4 antagonist; 75 μg in 50 μl PBS, n = 4) prior to bladder instillation completely blocked abdominal mechanical hypersensitivity induced by disulfide HMGB1 for all the filaments (Fig. 3b). No difference was detected between pre and post disulfide HMGB1 instillation (F = 0.028). On the other hand, disulfide HMGB1-induced abdominal mechanical hypersensitivity was not blocked when FPS-ZM1 (RAGE antagonist; 250 μg, n = 6) was infused into the bladder before disulfide HMGB1 bladder instillation (Fig. 3d). There were still significant differences between pre and post disulfide HMGB1 instillation for all von Frey filaments (Fig. 3d).

Systemic (intraperitoneally) treatment with either TLR4 antagonist TAK-242 (3 mg/kg, 30 min pretreatment, n = 5) [11] or RAGE antagonist FPS-ZM1 (10 mg/kg, 15 min pretreatment, n = 3) [12] before intravesical infusion of disulfide HMGB1 (10 μg) prevented HMGB1-induced abdominal mechanical hypersensitivity (Fig. 3c, e).

### Pretreatment of TLR4 and RAGE antagonists on micturition and histology

Intravesical disulfide HMGB1 (10 μg) did not change micturition volume or frequency (Table 1). No micturition changes were observed in groups pre-treated with TLR4 and RAGE antagonists either intraperitoneally (TLR4: 274 ± 42 in volume, 4.0 ± 0.7 in frequency; RAGE: 214 ± 37 in volume, 4.0 ± 0.6 in frequency) or intravesically (TLR4: 231 ± 16.9 in volume, 4.0 ± 0.5 in frequency; RAGE: 221 ± 19.4 in volume, 4.8 ± 0.3 in frequency) followed by intravesical disulfide HMGB1 when compared to disulfide HMGB1 instillation only group (264 ± 25.5 in volume; 4.0 ± 1.0 in frequency).

Pretreatment, either i.p. or intravesically, with TLR4 or RAGE antagonist did not elicit bladder inflammation (score = 0). Pretreatment with HMGB1 antagonist (i.p. or intravesical) followed by intravesical disulfide HMGB1 (10 μg/150) also showed minimal increases (not statistically significant) in edema (TAK 242, i.p, score = 0.13 ± 0.13; intravesical, score = 0; FPS-ZM1, i.p., score = 0, intravesical, score = 0.08 ± 0.08) compared to vehicle control (score = 0) or compared to intravesical disulfide (10 μg/150 μl alone; 0.5+ 0.22) (Fig. 2b, c, e, f).

## Discussion

We recently reported that intravesical activation of urothelial PAR4 receptors resulted in release of urothelial HMGB1, which mediated bladder pain [10]. Release of urothelial adenosine triphosphate (ATP) and activation of transit receptor potential vanilloid 1 (TRPV1) are well-described mechanisms of bladder pain [16–19]. Whether HMGB1 elicits bladder pain through ATP and/or TRPV1 remains to be investigated.

The present study extends our earlier findings since we clearly demonstrate that HMGB1 infused into the bladder is capable of inducing abdominal mechanical hypersensitivity (an indirect index of bladder pain). Furthermore, the redox state of HMGB1 is important since only intravesical disulfide HMGB1 but not the all-thiol (reduced) form induced abdominal mechanical hypersensitivity.

We also examined physiological and histological changes in response to different doses and different redox forms of HMGB1 infused intravesically. Changes in micturition parameters were only observed with the highest dose of disulfide and all-thiol HMGB1 (20 μg; decreased micturition volume and increased frequency) whereas none of the lower doses had any effect on micturition (Table 1). In terms of histological changes, only the highest dose tested (20 μg) of either disulfide or all-thiol HMGB1 was able to elicit minimal bladder inflammation (Table 2), while minimal to mild bladder edema and subtle stromal reactive changes were present with all doses of either disulfide or all-thiol HMGB1. These histological findings are consistent with our previous publication that HMGB1 mediates bladder pain without overt bladder inflammation [10]. Similarly, intraplantar injection of HMGB1 at 10 and 20 μg caused paw withdrawal latency decrease as well as edema but only 20 μg HMGB1 elicited mild inflammation in hind paw [20]. Furthermore, our findings that disulfide HMGB1 mediates bladder pain in a model with no overt bladder inflammation extend the findings of Tanaka et al [5] who found that systemic HMGB1 antagonists could prevent bladder pain after chemical (cyclophosphamide) injury of the bladder but did not affect inflammatory changes in this chemical cystitis model.

Recent studies implicate HMGB1 in mediating pain both at the organ level and at the central nervous system level (for a review see Kato J & Svensson CI) [6]. Pain hypersensitivity was elicited when HMGB1 was injected into sciatic nerve and anti-HMGB1 treatment alleviated mechanical allodynia after injury, but the nociceptive signaling pathway is still unclear [21]. Thrombomodulin (HMGB1 sequester) treatment alleviates intraplantar injection HMGB1 induced mechanical hypersensitivity, indicating HMGB1’s peripheral effect in nociception [20]. As an endogenous inflammatory mediator, HMGB1 influences adjacent neurons and glia, which contributes to the development of neuropathic pain states [21]. One report showed an increase in HMGB1 re-distribution into cytoplasm of sensory neurons in dorsal root ganglion in a model of tibial nerve injury induced neuropathic pain [22]. In this model, systemic application of glycyrrhizin, a HMGB1 blocker, reversed the neuropathic pain [22]. TLR4 and RAGE receptors were shown to be mediating nociception differentially in peripheral tissue and nervous system while HMGB1 redox forms recognize their receptors respectively [11, 23–27]. TLR4 downregulation in spinal glial cells attenuates mechanical allodynia in a rat model of trinitrobenzene sulfonic acid induced chronic pancreatitis [28]. On the other hand, RAGE mRNA and protein were increased in dorsal root ganglia after tibial nerve injury and RAGE inhibition by neutralizing antibody reversed the pain related behavior [29]. There is also evidence that systemic or intrathecal HMGB1 neutralizing antibody or a specific antagonist can alleviate pain mediated by TLR4 or RAGE receptors, suggesting central effect of TLR4 and RAGE receptors [8, 20].

Two strategies were used in the current study to identify the HMGB1 receptor mediating the disulfide HMGB1-induced abdominal mechanical hypersensitivity. A dose of disulfide HMGB1 (10 μg) that produced no inflammation and only minimal edema was chosen for the intravesical infusion. Systemic pretreatment with either a TLR4 (TAK-242) or a RAGE (FPS-ZM1) antagonist blocked abdominal mechanical hypersensitivity induced by disulfide HMGB1. Since both of these antagonists cross the blood-brain barrier [12, 30], the effect may be due to antagonism of central TLR4 or RAGE receptors and these receptors mediate pain in other models [24, 25]. We applied antagonists intravesically to determine whether and which of these receptors mediated the effect of disulfide HMGB1 at the organ level. TLR4 and RAGE receptors are found in the urothelium [26, 31] and sacral DRGs [29, 32] also contain TLR4 and RAGE although whether they innervate the bladder is not known. Intravesical pretreatment with TLR4 antagonist prevented hypersensitivity caused by disulfide HMGB1 while RAGE antagonist did not. Taken together, these findings indicate that TLR4 receptors at the organ level are responsible for the abdominal mechanical hypersensitivity induced by bladder infusion of disulfide HMGB1.

Our results also indicate that RAGE receptors modulate the effects of intravesical infusion of HMGB1 by acting not at the organ (i.e. bladder level) but possibly at the central nervous system level, since systemic administration was effective in blocking abdominal mechanical hypersensitivity. This agrees with the observation by Tanaka, et al. [5] that systemic administration of an antibody to HMGB1 or a RAGE inhibitor blocked cyclophosphamide- induced bladder pain. In contrast, we show that a systemic TLR4 antagonist administered systemically prevented disulfide HMGB1-induced bladder pain while Tanaka reported that a systemic TLR4 inhibitor had no effect on CYP-induced bladder pain [5]. This discrepancy may be due to the different method used to elicit bladder pain. Disulfide HMGB1 resulted in only minimal histological changes in the bladder while CYP is a strong chemical irritant that results in severe inflammation and hemorraghic cystitis [4].

PBS/IC is a condition characterized by bladder pain (or discomfort), frequency and urgency with unclear etiology [33] and in the absence of obvious bladder pathology [34]. Our current findings showed that disulfide HMGB1 elicited pain may account for bladder pain observed in the absence of inflammation. We realize that our model using intravesical infusion of substances (PAR4-AP; HMGB1) at doses that cause pain without accompanying micturition or inflammation changes, focus only on one aspect of PBS/IC, namely pain, without addressing increased frequency and urgency commonly seen in PBS/IC. Still these rodent models are useful because they are capable of eliciting bladder pain as a primary effect and not secondary to significant injury and inflammation. As such, they are useful tools in investigating the physiology of bladder pain in health and disease.

**Fig. 4:**
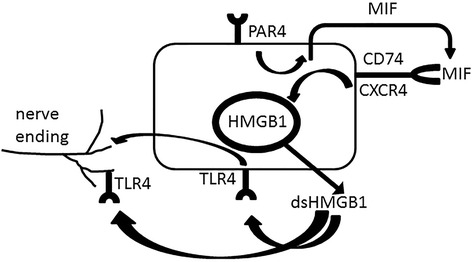
Role of HMGB1 in PAR4 induced bladder pain. Activation of PAR4 receptors on urothelial cells elicits release of urothelial macrophage migration inhibitory factor (MIF). MIF binds to urothelial MIF receptors (CD74/CXCR4) to mediate release of urothelial HMGB1. Disulfide HMGB1 (ds HMGB1) may bind to TLR4 receptors in urothelium and/or nerve terminal innervating the bladder to mediate bladder pain

It is possible that urine proteases, already elevated in PBS/IC patients, activate urothelial PAR4 receptors to release MIF into the urine [35, 36]. MIF, in turn, activates urothelial MIF receptors to elicit HMGB1 release. Oxidation of HMGB1 in the extracellular space [6] or in the urine results in disulfide HMGB1 that binds to either urothelial TLR4 receptors to induce further signaling resulting in bladder pain or may bind directly to the mucosa or possible to elicit bladder pain (Fig. 4). This schema remains to be validated in the clinical condition in future studies.

## Conclusions

We previously showed that activation of urothelial PAR4 receptors results in release of MIF and HMGB1 increasing abdominal mechanical hypersensitivity without bladder inflammation. We now report that HMGB1 infused directly into the bladder is capable to elicit mechanical hypersensitivity and this effect is produced by the disulfide isoform of HMGB1. Lastly, this effect is mediated by TLR4 receptors in the bladder and can also be modulated by systemic (presumably central) RAGE receptors. Neutralizing bladder MIF, MIF receptors, HMGB1 or antagonism of bladder TLR4 or systemic RAGE receptors may be potential specific and localized targets for bladder pain relief.

## Abbreviations

ATP: Adenosine triphosphate
CYP: Cyclophosphamide
H&E: Hematoxylin and eosin
HMGB1: High mobility group box-1
MIF: Macrophage migration inhibitory factor
PAR4: Protease activated receptor 4
PAR4-AP: PAR4-activating peptide
PBS: Phosphate buffered saline
PBS/IC: Bladder pain syndrome/interstitial cystitis
RAGE: Receptors for advanced glycation endproducts
TLR4: Toll- like receptor 4
TRPV1: Transit receptor potential vanilloid 1
VSOP: Voided stain on paper
